# Adiponectin and the risk of new-onset atrial fibrillation: a meta-analysis of prospective cohort studies

**DOI:** 10.1042/BSR20182284

**Published:** 2019-06-10

**Authors:** Ying Guo, Lixin Liu, Jianjun Wang

**Affiliations:** Department of Cardiology, The Affiliated Hospital of Jining Medical University, Jining 272049, China

**Keywords:** Adiponectin, Atrial fibrillation, Inflammation, Meta-analysis, Prospective cohort study

## Abstract

**Background:** Adiponectin has been suggested as a marker of many cardiovascular diseases. However, the association between serum adiponectin and incidence of atrial fibrillation (AF) in general population remains unclear. A meta-analysis was performed to systematically evaluate the potential influence of serum adiponectin at baseline on the incidence of AF during follow-up in general population.

**Methods:** Prospective cohort studies were identified via electronic search of PubMed and Embase databases. A randomized effect model was applied to combine the results. Predefined subgroup analyses were performed to evaluate the influence of study characteristics on the association between baseline adiponectin and risk of new-onset AF.

**Results:** Six cohort studies with 18558 community-derived participants were included, and 3165 AF cases were developed with a mean follow-up duration of up to 22 years. Meta-analysis showed that higher baseline circulating adiponectin was significantly associated with higher risk of new-onset AF during follow-up (hazard ratio [HR]: 1.17, 95% confidence interval [CI]: 1.08–1.27, *P*<0.001, *I^2^* = 52%). Subgroup analyses showed that the association between adiponectin and new-onset AF was significant in studies with mean follow-up duration over 10 years (five cohorts, HR = 1.22, *P*<0.001), but not in that with a follow-up duration < 10 years (one cohort, HR = 0.95, *P*=0.51; *P* for subgroup difference = 0.002).

**Conclusions:** Higher circulating adiponectin at baseline may be an independent risk factor for the development of new-onset AF during follow-up, particularly in cohort studies with longer follow-up durations.

## Introduction

Atrial fibrillation (AF) is one of the most common chronic arrhythmia which is associated with increased risk of stroke, heart failure (HF) and all-cause mortality [1]. The prevalence of AF was estimated at 33 million in 2015 all over the world. With the aging of global population, the prevalence of AF has been expected to increase 2.5-fold in the next 50 years [2]. Current treatments for AF mainly include rhythm control, rate control and anticoagulation [1,3]. Although catheter ablation to achieve pulmonary vein isolation may terminate AF in some patients, the recurrence remains high in long-term follow-up [3]. Therefore, identification of risk factors for the development of AF in general population is important for early prevention of AF and revealing of potential treatment targets.

Currently, established risk factors for AF include known cardiovascular diseases (CVDs), such as coronary artery disease (CAD), HF, and valvular heart disease, as well as conventional risk factors of CVDs, including aging, hypertension, diabetes mellitus (DM), and tobacco smoking [4]. Moreover, accumulating evidence suggests that insulin resistance and inflammation may also play important roles in the pathogenesis of AF [5,6]. Adiponectin, a cytokine generated by adipocytes, has been demonstrated to exert insulin-sensitizing, anti-inflammatory, and anti-atherogenic properties [7–10], which is suggested to serve as a potential biomarker for the risk of CVDs [11,12], including AF. Indeed, some cohort studies have been performed to evaluate the association between adiponectin and AF risk in community-derived general population [13–18]. However, results of these studies are inconsistent. Therefore, we aimed to perform a meta-analysis to comprehensively evaluate the association between baseline level of circulating adiponectin and incidence of AF in general population.

## Methods

We followed the MOOSE (Meta-analysis of Observational Studies in Epidemiology) [19] and Cochrane’s Handbook [20] guidelines during the design, implementation, analysis, and reporting for the present study.

### Database search

We searched the databases of PubMed and Embase for relevant records, using the terms ‘adiponectin’ and ‘atrial fibrillation’. We limited the search to studies in humans published in English language. The reference lists of original and review articles were also analyzed using a manual approach. The final literature search was performed on 10 July 2018.

### Study selection

Articles were included in the current meta-analysis if they met all the following criteria: (i) published as full-length article in English; (ii) reported as prospective cohort studies (regardless of sample size) with the follow-up duration of at least 1 year; (iii) included community-based adult population (≥18 years of age); (iv) circulating level of adiponectin was measured and identified as exposure of interest at baseline; (v) documented the incidences of AF during follow-up; (vi) reported the hazard ratios (HRs, at least adjusted for age and gender) and their corresponding 95% confidence intervals (CIs) of the risk of new-onset AF per 1-standard deviation (SD) increase in logarithmically transformed baseline adiponectin levels, or these data could be calculated. The diagnosis of new-onset AF was based on the definitions and criteria of the original articles. Reviews, letters, editorials, and studies with designs other than prospective cohort study were excluded from the current meta-analysis.

### Data extraction and quality evaluation

The processes of database searching, data extraction, and quality assessment were performed by two independent authors according to the predefined criteria. Discrepancies were resolved by discussion with the third author. Data that were extracted include: (i) study names, locations, and periods; (ii) characteristics of the participants (numbers, mean ages, and gender); (iii) forms and methods of adiponectin measurements; (iv) follow-up durations; and (v) outcomes (AF outcome assessment, numbers of cases with new-onset AF, and the variables adjusted when presenting the results); and (vi) primary data regarding the HRs and 95% CIs for the incidence of AF per 1-SD increase in logarithmically transformed adiponectin at baseline. The quality of each study was evaluated using the Newcastle–Ottawa Scale [21] which ranges from 1 to 9 stars and judges each study regarding three aspects: selection of the study groups; the comparability of the groups; and the ascertainment of the outcome of interest.

### Statistical analyses

Association between baseline adiponectin and risk of new-onset AF was presented as HRs and 95% CIs for the incidence AF per 1-SD increase in logarithmically transformed adiponectin, and the most adequately adjusted data were extracted. Data of HRs and their corresponding stand errors (SEs) were calculated from 95% CIs or *P* values, and were logarithmically transformed to stabilize variance and normalized the distribution [20]. We used the Cochrane’s Q test and *I^2^* test to evaluate the heterogeneity among the included cohort studies [22]. A significant heterogeneity was considered if *I^2^* > 50%. A random-effect model was applied to synthesize the HR data because this model is expected to retrieve a more generalized result via incorporation of the potential heterogeneity [20]. Sensitivity analyses, by removing individual study one at a time, were performed to evaluate whether the results of the meta-analysis was primarily driven by one influential study [23]. Predefined subgroup analyses were performed to evaluate whether the association between baseline adiponectin and risk of new-onset AF was affected by study characteristics such as sample sizes, mean ages, proportions of males, methods of adiponectin measurement, follow-up durations, AF incidences of the cohorts, and quality scores of the studies. Medians of the continuous variables were defined as the cut-off values for the stratification of the subgroups. In addition, potential publication bias was assessed by funnel plots with the Egger regression asymmetry test [24]. RevMan (Version 5.1; Cochrane Collaboration, Oxford, U.K.) and STATA software (Version 12.0; Stata Corporation, College Station, TX) were used for the meta-analysis and statistical analyses.

## Results

### Results of literature search

**Figure 1:**
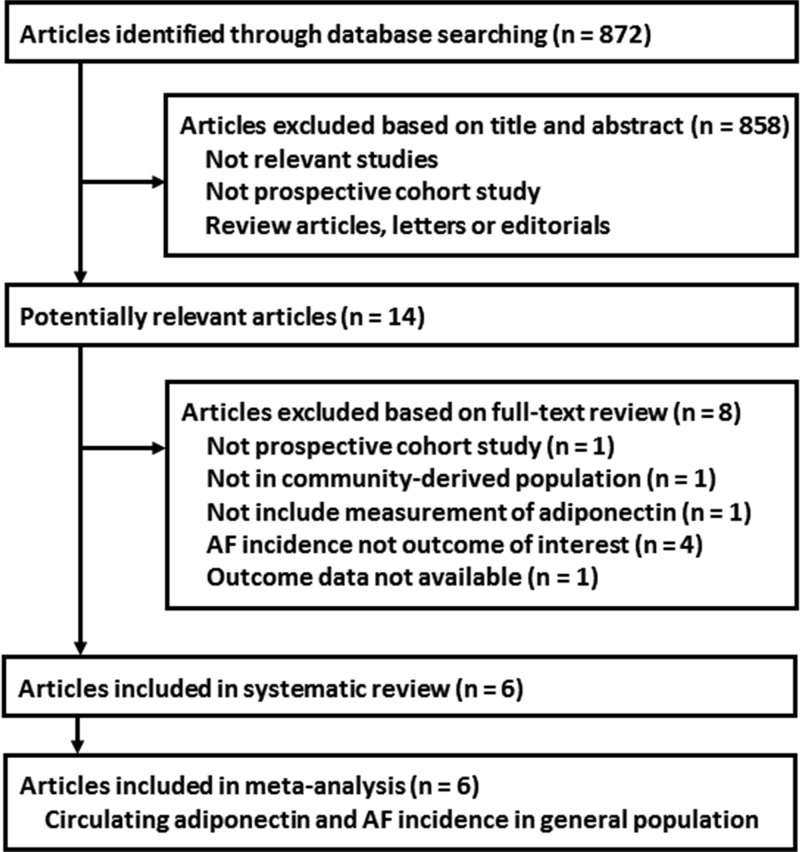
Flowchart of database search and study selection

The processes of literature search and study selection were presented in Figure 1. Briefly, 872 studies were obtained via initial literature search, and 858 were excluded via title and abstract screenings because they were irrelevant to the study purpose. The remaining 14 studies underwent full-text review. Of them, eight were further excluded because one of them was not a prospective cohort study, one did not include community-derived population, one study did not provide adiponectin level at baseline, four did not report AF incidence during follow-up, and the other one was without an available outcome data. Finally, six prospective cohort studies [13–18] were included.

### Study characteristics and quality evaluation

The characteristics of the included prospective cohort studies are presented in Table 1. Overall, our meta-analysis included 18558 community-derived participants from six cohort studies [13–18]. Four of them were performed in the U.S.A. [13,15–17], one in Australia [14], and the other one in Austria [18]. All the included studies reported the baseline levels of total adiponectin, while one of them also reported the level of high molecular weight (HMW) adiponectin [16]. Adiponectin was measured with enzyme-linked immunosorbent assay (ELISA) in four studies [13,14,16,18], while radioimmunoassay and multiplex assay were adopted in the other two studies respectively [15,17]. Confirmation of new-onset AF was made by electrocardiograph (ECG) and Holter in one study [13], while in the other five studies both ECG results and documented AF hospitalization were considered as new-onset AF events [14–18]. The follow-up durations varied from 7.6 to 20.0 years, and the incidences of AF in the included cohorts ranged from 8.0 to 27.8%. The Newcastle–Ottawa Scale varied from 8 to 9 in the included cohort studies.

**Table 1:** Characteristics of the included prospective cohort studies

Study	Design and location	Study periods	Number of participants	Mean age	Male	Adiponectin measurement	AF outcome assessment	Follow-up duration	AF cases	Adjusted factors	Quality score
		Year		Years	%			Years	*n* (%)		
2012 Framingham Offspring Study [13]	PC, U.S.A.	1999–2009	2487	61	46	Total, ELISA	ECG or Holter	7.6	206 (8.3)	Age, sex, BMI, SBP, treatment of hypertension, PR interval, clinically significant cardiac murmur, HF, and CRP	9
2014 Busselton Health Study [14]	PC, Australia	1995–2010	4267	52	47	Total, ELISA	Hospitalization of AF	15.0	343 (8.0)	Age, sex, height, hypertension treatment and BMI	8
2015 Cardiovascular Health Study [16]	PC, U.S.A.	1992–2009	3190	74 (>65)	36	Total and HMW, ELISA	ECG or hospitalization of AF	11.4	886 (27.8)	Age, sex, race, educational status, height, weight, SBP, treatment of hypertension, smoking, alcohol, self-reported health status, estimated GFR, NT-proBNP, subclinical CVD, DM, LDL-C, HDL-C, TG and hsCRP	9
2015 Health ABC Study [15]	PC, U.S.A.	1992–2013	2768	73 (70–79)	48	Total, RIA	ECG or hospitalization of AF	10.9	721 (26.0)	Race, age, sex, BMI, smoking, alcohol, statin treatment, hypertension, DM, CAD, HF and study site	9
2016 Women’s Health Initiative Study [17]	PC, U.S.A.	1993–2014	4937	66 (50–79)	0	Total, Multiplex assay	ECG or hospitalization of AF	11.1	892 (18.1)	Age, race, education, hypertension, DM, hyperlipidemia, CAD, HF, PAD, smoking, history of cancer and BMI	9
2017 Bruneck Study [18]	PC, Austria	1990–2010	909	59 (40–79)	50.7	Total, ELISA	ECG or Holter or hospitalization of AF	20.0	117 (12.9)	Age and sex	8

Abbreviations: BMI, body mass index; CRP, C-reactive protein; GFR, glomerular filtrating rate; HDL-C, high-density lipoprotein cholesterol; hsCRP, highly sensitive C-reactive protein; LDL-C, low-density lipoprotein cholesterol; NT-proBNP, N-terminal pro-B type natriuretic peptide; PAD, peripheral artery disease; PC, prospective cohort; RIA, radioimmunoassay; SBP, systolic blood pressure; TG, triglyceride.

### Association between adiponectin and new-onset AF

**Figure 2:**
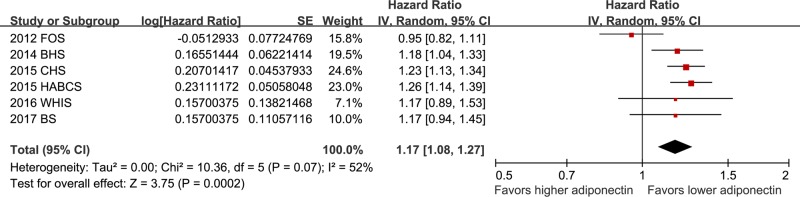
Forest plots for the meta-analysis of the association between adiponectin at baseline and subsequent risk of new-onset AF in general population Data were presented as HRs and 95% CIs for the incidence of AF per 1-SD increase in logarithmically transformed adiponectin at baseline.

Pooled results with a random-effect model showed that higher baseline circulating adiponectin was significantly associated with higher risk of new-onset AF during follow-up (HR: 1.17, 95% CI: 1.08–1.27, *P*<0.001; Figure 2) with considerable heterogeneity (*P* for Cochrane’s Q test =0.07, *I^2^* = 52%). Sensitivity analyses by excluding one study at a time did not change the results (HR: 1.14–1.22, *P* all <0.05; Table 2). Heterogeneity significantly reduced (*P* for Cochrane’s Q test =0.92, *I^2^* = 0%) after excluding the Framingham Offspring Study [13], indicating that this study is the major contributor of heterogeneity of the meta-analysis.

**Table 2:** Sensitivity analyses

Studies excluded	HR (95% CI)	*I^2^*	*P* for heterogeneity	*P* for outcome effect
2012 Framingham Offspring Study [13]	1.22 (1.16, 1.29)	0%	0.92	<0.001
2014 Busselton Health Study [14]	1.16 (1.05, 1.29)	61%	0.03	0.005
2015 Cardiovascular Health Study [16]	1.15 (1.03, 1.27)	58%	0.05	0.01
2015 Health ABC Study [15]	1.14 (1.03, 1.26)	53%	0.08	0.009
2016 Women’s Health Initiative Study [17]	1.17 (1.07, 1.28)	61%	0.03	<0.001
2017 Bruneck Study [18]	1.17 (1.06, 1.28)	61%	0.04	0.001

### Subgroup analyses

Subsequent results of subgroup analyses indicated that study characteristics, such as sample sizes of the cohorts, mean ages of the participants, gender, methods of adiponectin measurements, AF incidences of the study cohorts, or the quality scores of the studies, did not significantly affect the association between adiponectin and new-onset AF (*P* for subgroup differences, all >0.05, Table 3). However, the association between adiponectin and new-onset AF was significant in studies with mean follow-up duration over 10 years (five cohorts, HR = 1.22, 95% CI: 1.16–1.29, *P*<0.001), but not in that with a follow-up duration < 10 years (one cohort, HR = 0.95, 95% CI: 0.82–1.11, *P*= 0.51; *P* for subgroup difference =0.002; Table 3).

**Table 3:** Subgroup analyses

Variables and cutoff	Number of studies	HR (95% CI) for subgroup	*I^2^*	*P* for subgroup effect	*P* for subgroup difference
Sample sizes					
<3000	3	1.12 [0.93, 1.35]	79%	0.23	
≥3000	3	1.21 [1.13, 1.30]	0%	<0.001	0.46
Mean ages (years)					
<65	3	1.09 [0.94, 1.27]	61%	0.24	
≥65	3	1.24 [1.16, 1.32]	0%	<0.001	0.12
Male (%)					
<40	2	1.22 [1.12, 1.33]	0%	<0.001	
≥40	4	1.14 [1.01, 1.29]	68%	0.04	0.36
Adiponectin measurements					
ELISA	4	1.13 [1.01, 1.27]	64%	0.03	
Others	2	1.25 [1.14, 1.37]	0%	<0.001	0.20
AF confirmation					
Include AF hospitalization	5	1.22 [1.16, 1.29]	0%	<0.001	
Not include AF hospitalization	1	0.95 [0.82, 1.11]	—	0.51	0.002
Follow-up duration (years)					
<10	1	0.95 [0.82, 1.11]	—	0.51	
≥10	5	1.22 [1.16, 1.29]	0%	<0.001	0.002
AF incidence (%)					
<15	3	1.09 [0.94, 1.27]	61%	0.24	
≥15	3	1.24 [1.16, 1.32]	0%	<0.001	0.12
Quality scores					
=8	2	1.18 [1.06, 1.31]	0%	0.003	
=9	4	1.16 [1.02, 1.31]	71%	0.02	0.83

### Publication bias

**Figure 3:**
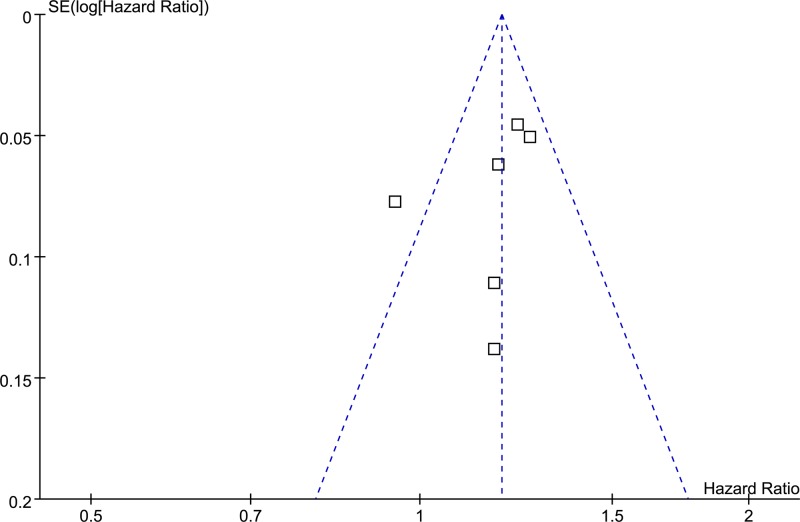
Funnel plots for the meta-analysis of the association between adiponectin and subsequent risk of new-onset AF during follow-up in general population

The funnel plots regarding adiponectin at baseline and the risk of AF during follow-up is shown in Figure 3. The funnel plot was symmetrical on visual inspection. Results of Egger regression test suggested that no significant publication bias was detected (*P*=0.33).

## Discussion

In the present study, by pooling the results of all available prospective cohort studies, results of our meta-analysis showed that higher circulating adiponectin at baseline is independently associated with increased risk of AF incidence in general population. Subgroup analyses indicated that the association between adiponectin and AF incidence is significant in cohorts with longer follow-up durations (≥10 years), but not in studies with shorter follow-up durations (<10 years). These findings are paradoxical to previous notion that adiponectin may be a protective factor against AF incidence since it is an anti-inflammatory factor.

To the best of our knowledge, our study is the first meta-analysis that evaluates the association between circulating adiponectin at baseline and the risk of AF in general population. We found that higher adiponectin at baseline appeared to be an independent risk factor for AF during follow-up. Although findings from experimental studies demonstrated that adiponectin, via exerting its insulin-sensitizing, anti-inflammatory, and anti-atherogenic properties, may be cardioprotecive, findings from epidemiological studies regarding the association between adiponectin and CVDs showed different results. In a meta-analysis of 24 prospective studies, circulating adiponectin at baseline was found to have no significant association with CAD incidence [25]. Moreover, in patients with established CVDs, higher adiponectin was independently associated with increased risk of CAD recurrence [25]. These findings were further confirmed in a subsequent meta-analysis which showed that adiponectin was associated with increased mortality in patients with already established CVDs [26]. Similarly, increased serum adiponectin was related to an elevated risk of ischemic stroke in a previous meta-analysis of 17 prospective studies with a total of 23717 participants [27]. Moreover, accumulating evidence from prospective cohort studies suggests that higher serum adiponectin may be an independent risk factor for HF incidence in community-based population [28,29]. Our results expanded these findings by showing that higher serum adiponectin was associated with increased risk of AF in general population. These findings further added the potential complexity regarding the association between adiponectin and CVDS. In fact, the potential role of adiponectin in patients with established AF was also controversial according to previous studies. In a prospective study including 874 patients with paroxysmal AF, high circulating adiponectin is independently associated with AF recurrence after catheter ablation [30]. However, another study showed that low plasma adiponectin was significantly associated with major cardiovascular events in female anticoagulated patients with nonvalvular AF [31]. Moreover, adiponectin was found to be inversely associated with the degree of platelet activation and risk of stroke in anticoagulated patients with AF, indicating a potential protective effect of higher adiponectin against stroke incidence in AF patients [32]. Taken together, although current findings suggest that increased adiponectin may be a marker of higher risk for AF development in general population, the influence of adiponectin on the pathogenesis, progression, and prognosis of AF may be much more complicated. Moreover, the potential mechanisms involved are largely unknown. Further studies are needed to clarify the exact relationship between adiponectin and AF.

Sensitivity analyses and subgroup analyses indicated that the Framingham Offspring Study [13] is the major contributor to the heterogeneity of the meta-analysis. Of note, the mean follow-up duration in the Framingham Offspring Study is shorter than others, suggesting that the association between adiponectin and AF incidence is significant in cohorts with longer follow-up durations (≥10 years), but not in studies with shorter follow-up durations (<10 years). Moreover, AF incidence was identified by the results of ECG and Holter in the Framingham Offspring Study [13], while other studies also included AF hospitalization as an indicator of AF incidence. These differences in the confirmation of AF incidence may also contribute to the heterogeneity. However, results of subgroup analyses regarding the potential influence of follow-up duration on the association between adiponectin and AF risk should be interpreted with caution since limited number of studies were included in each stratum.

Our study has limitations. First, adiponectin was measured via different methods in the included studies, and the strategies for the detection of new-onset AF varied. These may confound the result and contribute to the heterogeneity. In addition, as mentioned above, the results of subgroup analyses should be interpreted cautiously because limited studies were available and the results were based on data of study-level rather than individual patient-level. Moreover, a dose–response association between circulating adiponectin at baseline and the AF risk was unable to determine, because limited cohorts were included. Besides, as a common limitation of meta-analyses of observational studies, the association between adiponectin and AF incidence may be confounded by residual factors that were unadjusted when presenting the results. Finally, the clinical relevance of the findings of the meta-analysis should be investigated in the future. Currently, we were unable to determine whether the relationship between higher adiponectin and increased risk of AF incidence is causative. Future studies are needed to elucidate the potential mechanisms underlying the association between higher adiponectin and increased risk of AF incidence.

In conclusion, our meta-analysis indicated that higher circulating adiponectin at baseline may be an independent risk factor for the development of new-onset AF during follow-up, particularly in cohort studies with longer follow-up durations. Whether increased adiponectin is a surrogate marker or a therapeutic target during the pathogenesis of AF in general population deserves further investigation.

## Abbreviations

AF: atrial fibrillation
CAD: coronary artery disease
CI: confidence interval
CVD: cardiovascular disease
ECG: electrocardiograph
HF: heart failure
HR: hazard ratio
SD: standard deviation
